# Companion of choice at birth: factors affecting implementation

**DOI:** 10.1186/s12884-017-1447-9

**Published:** 2017-08-31

**Authors:** Tamar Kabakian-Khasholian, Anayda Portela

**Affiliations:** 1 Health Promotion and Community Health Department, Faculty of Health Sciences, American University of Beirut, Beirut, Lebanon; 2 Department of Maternal, Newborn, Child and Adolescent Health, World Health Organization, Geneva, Switzerland

**Keywords:** Companion of choice at birth, Continuous support during childbirth, Labour companionship, Lay companion, Doula, Implementation

## Abstract

**Background:**

Two recent recommendations made by the World Health Organization confirm the benefits of companion of choice at birth on labour outcomes; however institutional practices and policies do not always support its implementation in different settings around the world. We conducted a review to determine factors that affect implementation of this intervention considering the perspectives and experiences of different stakeholders and other institutional, systemic barriers and facilitators.

**Methods:**

Forty one published studies were included in this review. Thirty one publications were identified from a 2013 Cochrane review on the effectiveness of companion of choice at birth. We also reviewed 10 qualitative studies conducted alongside the trials or other interventions on labour and birth companionship identified through electronic searches. The SURE (Supporting the Use of Research Evidence) framework was used to guide the thematic analysis of implementation factors.

**Results:**

Women and their families expressed appreciation for the continuous presence of a person to provide support during childbirth. Health care providers were concerned about the role of the companion and possible interference with activities in the labour ward. Allocation of resources, organization of care, facility-related constraints and cultural inclinations were identified as implementation barriers.

**Conclusion:**

Prior to introducing companion of choice at birth, understanding providers’ attitudes and sensitizing them to the evidence is necessary. The commitment of the management of health care facilities is also required to change policies, including allocation of appropriate physical space that respects women’s privacy. Implementation research to develop models for different contexts which could be scaled up would be useful, including documentation of factors that affected implementation and how they were addressed. Future research should also focus on documenting the costs related to implementation, and on measuring the impact of companion of choice at birth on care-seeking behavior for subsequent births.

## Background

Companion of choice at birth is defined as the continuous presence of a support person during labour and birth [1]. The intervention has been recommended by the World Health Organization (WHO) to improve labour outcomes and women’s satisfaction with care [2, 3]. It has also been identified as a key element in the WHO vision of quality of care for pregnant women and newborns [4]. Different names have been given to the intervention including continuous support during childbirth, companion of choice at birth, labour companion, emotional support during birth. We will refer to this intervention herein as companion of choice at birth.

Evidence for companion of choice at birth emanates from a Cochrane systematic review conducted in 2013 [1] showing that companion of choice at birth increases the likelihood of vaginal births, therefore reduces the need for caesarean sections and the use of forceps or vacuum during vaginal births. In addition, it reduces the need to use pain medications during labour, it shortens the duration of labour and improves women’s satisfaction with care. It also improves Apgar scores of the newborns.

The Cochrane systematic review included 22 trials involving more than 15,000 women. The trials were conducted in low, middle and high-income countries with considerable variation in hospital settings. The form of care evaluated was continuous presence and support during labour or labour and birth. The control group received “usual care” as defined by the study investigators that did not involve continuous support during labour or during labour and birth.

In the research conducted, the support person varied from a nurse or midwife of the hospital staff to a doula, a woman not related to the labouring woman and not a hospital staff but employed through the trial to accompany labouring women, to a companion of choice identified by the pregnant woman from her social network. The most beneficial form of support appears to be from a person who is not a member of the woman’s social network, is not hospital staff and who has some experience or has received some informal training. However, in the absence of such a person, the support from a person of choice from among the woman’s family or friends improves women’s satisfaction with care [1].

Institutional routines and policies, providers’ attitudes as well as the physical environments of hospitals do not always support the implementation of the intervention. Given the multiple dimensions of this intervention including variation in the delivery of the intervention according to the context and the provider of the support, there is a need for greater understanding of the factors that influence successful implementation and sustainability of the intervention. These factors include the perspectives, experiences, knowledge and skills of different stakeholders involved in implementation, as well as relevant decision making processes, and barriers and facilitators within and outside of the health facility.

This paper aims at a) describing stakeholder perspectives and experiences with the intervention; b) identifying the barriers and facilitating factors to implementation; and c) describing those implementation factors which could be linked to programmes who reported on increased women’s satisfaction.

## Methods

This is a literature review of factors influencing implementation of companions of choice at birth. It is based on information describing the implementation of the intervention derived from trials identified through the Cochrane review [1], qualitative research conducted alongside any of these trials, or studies describing health care providers’ attitudes towards the provision of support during childbirth.

This review included 41 publications. We assessed all the studies identified in the Cochrane review in 2013 [1] for reported information on implementation factors. These also included studies that were excluded from the Cochrane review [1]. We included 31 studies in our review through this process:22 published articles [5–26] included in the meta-analysis of the Cochrane Review [1]and 9 excluded from it [27–35]. We excluded one of the trials included in the Cochrane review [1] because it was not published in English [36]. In addition, we conducted electronic searches on Pubmed and Medline to identify qualitative studies describing the experiences of stakeholders with the implementation of companion of choice at birth published through March 2015. We also searched the bibliographies of the published trials to identify qualitative studies on implementation factors or feasibility of the intervention published alongside the trials. We identified an additional 10 studies in this category [37–46].

No quality assessment of studies was done for this review, considering that we were interested in identifying publications reporting on implementation factors that were not directly related to validity of the outcomes assessed in the trials.

The first author extracted data from the publications included in this review, and used the SURE (Supporting the Use of Research Evidence) framework [47] to guide the extraction and categorization of information on implementation factors into meaningful themes. A research assistant verified the coding and abstracting of the data. Thematic analysis was used to identify factors that influence implementation of the intervention as well as different stakeholder perspectives and experiences of the intervention and contextual characteristics. To capture information about the context of the implementation in the reviewed studies, we retrieved information from the discussion sections. The variation in settings in these studies between high, middle and low income countries allowed for some comparison of factors in the different contexts.

## Results

The characteristics of the studies included in this analysis are presented in Table 1. Among all the reviewed studies, only eight aimed at reporting on implementation factors for the introduction of a companion of choice at birth in a hospital [13, 23, 27, 32, 43–46]. The remaining studies in this review aimed at reporting the effectiveness of the practice.

**Table 1:** Table of characteristics of included studies for companion of choice at birth

	Study	Study design	Setting	Description of Intervention	Specific study outcomes considered in this review
1	Alexander et al., 2014	Mixed-methods study	Ghana	No intervention	Women’s views about social support during childbirth
2	Al-Mandeel et al., 2013	Prospective cohort study	Kingdom of Saudi Arabia	No intervention	Women’s preferences and attitudes towards companions during childbirth
3	Banda et al., 2010	Pre-post intervention survey	Malawi	Support during labour by lay companions arriving at the hospital with labouring women was introduced in the hospital.	Women’s experiences
4	Breart et al., 1992	RCT	Belgium, France, Greece	Permanent presence of a midwife compared to varying degrees of presence. Fathers were allowed to be present	
5	Bruggerman et al., 2007	RCT	Brazil	Support was ‘presence of a chosen companion during labour and delivery’. Companions received verbal and written information to orient on their role. In 47.6% of the sample the woman’s companion was her partner, for 29.5% it was her mother. The control group received care where a companion during labour and birth was not permitted.	Satisfaction with labour and delivery
6	Brown et al., 2007	RCT	South Africa	Hospitals in the intervention group were given training and access to the WHO Reproductive Health Library. A multidimensional educational package was implemented at the intervention hospitals over 2 months	
7	Campbell et al., 2006	RCT	USA	Continuous support by an additional support person (doula group), was compared with women who did not have this additional support person (control group). The doula group had two two-hour orientation sessions about labour support. Control group had support people of their own choosing.	
8	Campbell et al., 2007	RCT	USA	Same trial as above but this secondary analysis focused on maternal perceptions of infant, self and support from others at 6–8 weeks postpartum.	Satisfaction with care
9	Campero et al., 1998	Qualitative postpartum interviews	Mexico	Mothers receive psychosocial support from a doula, compared with women without a doula, who gave birth following normal hospital routine. In the former group, doulas were incorporated into the labour and delivery rooms and introduced to physicians and nurses, and their responsibilities in accompanying the pregnant women were explained to hospital staff.	Mother’s view of their experience
10	Cogan et al., 1988	RCT	USA	Support provided by a Lamaze childbirth preparation instructor. Support included continuous presence, acting as a liaison with hospital staff, providing information, and teaching relaxation and breathing measures to the woman and a present family member. Usual care: intermittent nursing care. Family members were allowed to be present.	
11	Dickinson et al., 2002	RCT	Australia	Group 1 –continuous physical and emotional support by midwifery staff, and women were encouraged manage their labour with the assistance of a midwife with the intention of avoiding epidural analgesia. Group 2 –continuous midwifery support was not provided and women were encouraged to have epidural analgesia as their primary method of pain relief in labour The women in each of the two groups were at liberty to choose an alternative form of analgesia at any time.	
12	Dickinson et al., 2003	RCT and post survey	Australia	Same intervention and control group as above but post survey conducted 6 months postpartum The women in both groups were at liberty to choose an alternative form of analgesia at any time.	Maternal satisfaction of childbirth
13	El-Nemer et l, 2006	Qualitative	Egypt	No intervention	Women’s experiences of hospital care
14	Gagnon et al., 1997	RCT	Canada	Intervention group: 1-to-1 care consisting of a nurse during labour and birth who provided emotional support, physical comfort, and instruction for relaxation and coping techniques. Care was provided by on-call nurses who were hired specifically for the study and had received a 30-h training program and quarterly refresher workshops. Training included critical reviews of the literature concerning the effects of the intrapartum medical and nursing practices, as well as discussions of stress and pain management techniques. Control group received usual nursing care by the regular unit staff, consisting of intermittent support and monitoring.	
15	Gagnon et al., 1999	Secondary analysis of RCT	Canada	Same intervention and control groups as above	
16	Gordon et al., 1999	RCT	USA	Support provided by a trained doula. The control group received “usual care” which did not include the support of a doula. The partner was present in 80% of all birth included in the study.	Mothers’ evaluations of their experiences
17	Hemminki et al., 1990	RCT	Finland	Reports on a pilot study with support provided by lay woman to labouring women arriving to one hospital without their male partners. Reports also on three trials testing 1:1 support by midwifery students from enrolment until transfer to the postpartum ward. The midwifery students volunteered, were not specially trained in support and responsible for the other routine intrapartum care. The control group was ‘cared for according to the normal routine of the midwife and by a medical student, if s(he) was on duty’. Over 70% of fathers were present.	Mothers’ evaluations of their experiences
18	Hodnett et al., 1989	RCT, stratified by type of prenatal classes (Lamaze vs general)	Canada	Support provided by a community ‘lay’ midwife or midwifery apprentice and included physical comfort measures, continuous presence, information, emotional support, and advocacy. The control group received usual hospital care which includes the intermittent presence of a nurse. All but 1 woman also had husbands or partners present during labour. Support began in early labour at home or in hospital and continued through delivery.	Women’s perceived control during childbirth
19	Hodnett et al., 2002	Multi-centre RCT with prognostic stratification for parity and hospital	Canada, USA	Continuous support from staff labour and delivery nurses who had volunteered and received a 2-day training workshop in labour support. The nurses with training were part of the regular staffing complement of the unit. Control group received intermittent support from a nurse who had not received labour support training	Birth experience and future preferences for labour support
20	Hofmeyr et al., 1991	RCT	South Africa	Support by carefully trained, volunteer lay women, for at least several hours (supporters not expected to remain after dark). Control group received intermittent care on a busy ward. Husbands/family members were not permitted	Mothers’ perceptions of labour
21	Kabakian-Khasholian et al., 2015	Qualitative	Egypt, Lebanon, Syria	No intervention	Women’s and health care providers perceptions about labour companionship
22	Kashanian et al., 2010	RCT	Iran	Support provided by an experienced midwife in an isolated room. Midwife-led support included close physical proximity, touch, and eye contact with the labouring women, and teaching, reassurance, and encouragement. The midwife remained with the woman throughout labour and delivery, and applied warm or cold packs to the woman’s back, abdomen, or other parts of the body, as well as performing massage according to each woman’s request. Control group included women admitted to the labour ward (where 5–7 women labour in the same room), did not receive continuous support, and followed the routine orders of the ward. They did not have a private room, did not receive one-to-one care, were not permitted food, and did not receive education and explanation about the labour process. The only persons allowed in the delivery room were nurses, midwives, and doctors.’	
23	Kennel et al., 1991	RCT + retrospective non-random control group	USA	Continuous support provided by a trained doula during labour and birth. Observed group received the routine intermittent presence of a nurse and continuous presence of an ‘inconspicuous observer’ who ‘kept a record of staff contact, interaction and procedures’. The observer was away from the bedside and never spoke to the labouring woman. A retrospective non-random control group was used.	
24	Klaus et al., 1986	RCT	Guatemala	Continuous emotional and physical support by a lay doula with no obstetric training. Control group: usual hospital routines (described as no consistent support)	
25	Kopplin et al., 2000	RCT	Chile	Psychosocial support during labour from a companion chosen by the pregnant woman. The companions were trained by trial staff to provide emotional support, promote physical comfort and encourage progress of labour, without interfering with the activities of the obstetricians or midwives. They were with the labouring woman continuously from admission to delivery. Women were encouraged to pick a companion who had experienced a vaginal birth. Control group did not have companion. Both groups laboured in a room with other women where curtains were pulled for privacy	Experience with childbirth
26	Kumbani et al., 2013	Qualitative	Malawi	No intervention	Women’s perception of perinatal care
27	Langer et al., 1998	RCT	Mexico	Continuous support from 1 of 10 retired nurses who had received doula training throughout labour, birth, and the immediate postpartum period. Support included emotional support, information, physical comfort measures, social communication, ensuring immediate contact between mother and baby after birth, and offering advice about breastfeeding during a single brief session postnatal. Control group: women received ‘routine care’.	Satisfaction
28	Lindow et al., 1998	RCT	South Africa	The intervention group received oxytocin followed by routine monitoring by labour ward midwives in addition to the presence of a support person for 1 h. The support persons were from among the hospital cleaning staff from the same racial and linguistic group as the woman. They provided encouragement. The control group received oxytocin and routine monitoring of labour ward midwives.	
29	Madi et al., 1999	RCT	Botswana	Continuous presence of a female relative (usually her mother) in addition to usual hospital care Control group: usual hospital care, which involved staff: patient ratios of 1:4, and no companions permitted during labour	
30	Maimbolwa et al., 2001	Mixed-methods	Zambia	No intervention	Women’s and health care providers perspectives about labour companionship in hospitals.
31	Manning-Orenstein, 1998	Non-randomized intervention	USA	Women chose between a doula support group or a Lamaze birth preparation group.	
32	McGrath et al., 2008	RCT	USA	Support consisted of a doula who met the couple or woman at the hospital as soon as possible after Random assignment (typically within an hour of their arrival at the hospital) and remained with them throughout labour and delivery. Doula support included continuous bedside presence during labour and delivery, although her specific activities were individualised to the needs of the labouring woman. Other support included close physical proximity, touch, and eye contact with the labouring woman, and teaching, reassurance, and encouragement of the woman and her male partner. All doulas completed training requirements that were equivalent to the DONA International doula certification Control group: routine obstetric and nursing care which included the presence of a male partner or other support person	Perceived experience with the doula
33	McGrath et al., 1999	RCT	USA	The Epidural group received epidural analgesia, the control group received narcotic medication followed by epidural analgesia if necessary. The doula group received continuous doula support with narcotic or epidural analgesia if necessary.	

All identified implementation factors are presented in Table 2.

**Table 2:** Acceptability, appropriateness and feasibility of companion of choice at birth

Level	Factors affecting implementation	Studies
Women and their families	a. In general, women and their male partners, when present, appreciated the presence of a support person with them, whether that person was a doula or a student nurse.	a. McGrath & Kennell, 2008; Hemminki, 1990; Hodnett et al., 2002
	b. Women had low expectation in the quality of care, they and their families reported experiencing bad treatment at the hospital.	b. Brown et al., 2007; Madi et al., 1999; Kabakian-Khasholian et al., 2015.
	c. Women expressed some reservations about lay female companions from the community in the form of fear of being exposed to the companion and the consequent gossip expected in the community, therefore a worry to keep up with social expectations.	c. Al-Mandeel et al., 2013; Maimbolwa et al., 2001; Alexandre et al., 2013.
	d. Lay female companions who were not related to women were viewed as an ally with no interest in the hospital system and easy to communicate with.	d. Hofmeyer et al., 1991; Campbell et al., 2007
	e. Male partners presence was appreciated for providing emotional and spiritual reassurance to women and to witness the challenges of childbirth endured by women however, they were viewed as unskilled, not usually present throughout labor and less interactive compared to female companions. Women feared that their male partners could not handle the process of labour and birth. In some cultures, the presence of the male partners is not socially acceptable.	e. Kennell et al., 1991; McGrath and Kennell, 2008; Morhason-Bello et al., 2009; Scott et al., 1999; Mosallam et al., 2004; Kabakian-Khasholian et al., 2015; Qian et al., 2001; Alexandre et al., 2013.
Health care providers	a. Health care providers were found to lack the positive attitudes of providing emotional and spiritual support as labour support was not considered a professional task.	a. Hemminki et al., 1990; Morhason-Bello et al., 2009
	b. Nurse and midwives sometimes were apprehensive about the presence of a support person and not quite sure about the effect on the cooperation of women with the staff or the skills of the lay person or their fear from the use of traditional medicine.	b. Banda et al., 2010; Maimbolwa et al., 2001; Cogan & Spinnato, 1988; Hemminki et al., 1990; Qian et al., 2001.
	c. The presence of the lay companion was viewed positively in reducing the dependency on the overloaded staff. Providers were perceived to be friendlier in the presence of the companion.	c. Madi et al., 1999; Bruggeman et al., 2007; Maimbolwa et al., 2001
	d. Health care providers should be informed of the evidence and motived by the high patient satisfaction with the implementation of this practice.	d. Bruggeman et al., 2007; Campbell et al., 2006
Other stakeholders	Community agencies including local health departments and health care providers can act as a source of information for doulas.	Campbell et al., 2007
Health system factors	a. The presence of the companion during childbirth might improve accessibility to health services in subsequent births through the reduction of the negative effects of the hospital environment in terms of reducing the feelings of being left alone, insecurity and neglect.	a. Langer et al., 1998; Maimbolwa et al., 2001; Kumbani et al., 2013
	b. Identified implications on human resources include: freeing the time of nurses and midwives thus improving quality of care, organization at the ward to change shift for	b. Madi et al., 1999; Yuenyong et al., 2012; Brown et al., 2007; Maimbolwa et al., 2001; Scott et al., 1999; Gordon et al., 1999
	c. nurses and midwives, the availability of the doula early during labour.	c. Langer et al., 1998; Kashanian et al., 2010; Banda et al., 2010; Kabakian-Khasholian et al., 2015, Campbell et al., 2007; Qian et al., 2001
	d. Training and hiring of nurses or midwives as doulas could be considered in some settings. Clear communication and some training about the duties of the lay companion are expected in others. Retired nurses hired as companions could be desensitized to women’s needs.	d. Yuengyong et al., 2012; Brown et al., 2007; Kabakian-Khasholian et al., 2015, Maimbolwa et al. 2001; Qian et al., 2001.
	e. Some implications on facilities include: availability of lounges for lay companions short breaks, privacy in labour rooms, space for companions in labour rooms.	
Social and political factors	a. Changes to national, state or hospital policies against having lay companions during childbirth might be necessary.	a. Madi et al., 1999; Kabakian-Khasholian et al., 2015; Yuengyong et al., 2012; Brown et al., 2007
	b. The cost of hiring a doula or the transportation need to be considered, however in general, having a lay companion or a doula is likely to result in overall reduction of costs in obstetric interventions and complications.	b. Madi et al., 1999; Campbell et al., 2007; Brown et al., 2007; Scott et al., 1999; Gordon et al., 1999
	c. Sustainability is believed to result from political commitment at the state and hospital level and from raising public awareness.	c. Brown et al., 2007

Categories adapted from: The SURE Collaboration, 2011

### Stakeholders’ perspectives

Studies mainly reported on the experiences and perspectives of women and health care providers. Receiving support by a companion of choice at birth was reported as a positive experience that was largely appreciated by women, and when present, by their male partners [13–15]. In three other studies, the presence of the companion was experienced as a buffer to women’s and their families’ low expectations and experiences of bad treatment at hospitals [16, 27, 43].

In some close-knit communities, where most people knew each other, women were reserved about the presence of doulas recruited from their same community or the presence of a female family member with them during childbirth [39, 42, 45]. They expressed their worries of being exposed to the companion and the consequent expected gossip in the community about them not being able to keep up with social expectations of behaviour, such as not losing control and not shouting. This is in contrast to another low-income community where lay female companions were regarded as allies not invested in the hospital system and their presence was appreciated [17].

In three studies conducted in high-income countries and one from a low-income setting, the presence of a female companion was perceived to be beneficial in addition to the presence of male partners. Despite the fact that male partners were appreciated for providing emotional and spiritual reassurance, women perceived them as lacking the skills needed for other aspects of support necessary to them [14, 18, 32, 45] as well as not coping well with seeing women go through labour and birth [45]. Both in low and high income country settings, male partners were found to be not consistently present throughout labour, they were mainly absent in early and late labour and were viewed as being less physically interactive compared to female companions [32, 45]. In conservative cultures, such as in Arab countries, in Ghana, and in China, women sometimes appreciated the presence of the male partner to witness the challenges that women go through during childbirth [43–45]. In Arab cultures, the presence of the male partners was not socially acceptable especially in shared labour rooms [40, 43].

The attitudes of health care providers involved in the intervention were sometimes positive as they perceived the presence of the companion helpful in reducing the dependency of women on the staff. This was mainly important in settings with shortage of nursing and midwifery staff [16, 21, 39]. The presence of the companion was noted to positively influence the behaviour of the staff towards women [21]. Some studies reported negative attitudes and some resistance in acceptance of companions to labour wards [13, 20, 39, 44, 46]. Nurses and midwives reported their doubts about the role of the companion and expected less cooperation of women with the staff throughout labour and birth in the presence of the companion [13, 20, 39, 44, 46]. Concerns were reported about the use of traditional medicines by lay companions [39]. Providers also reported concerns about companions who are not part of the hospital staff, interfering in medical decisions and about cross infections in the labour and delivery ward [39, 43, 44]. Efforts such as influencing the attitudes of health care providers by informing them about the evidence on companion of choice at birth and motivating them by sharing positive birth experiences are considered to be necessary for the successful implementation of this practice [21, 22].

### Barriers and facilitating factors for implementation

A number of health service-related barriers and facilitating factors were identified in the implementation of companion of choice at birth in hospital settings. These varied according to resources available in the facility and to the people providing support. Whereas having a female relative as a companion is considered a low cost intervention that is valued in facilities with shortage of nursing staff [16, 25, 39], the task of providing continuous support by nurses and midwives of the hospital was reported to intensify the human resource shortages [39] or had implications on the organization of shifts at the labour ward [27]. Where doulas were considered as labour or birth companions, their arrival to the labour ward early during labour was deemed necessary, for example before the woman had taken the decision to ask for an epidural [28].

The presence of an accompanying person was also found to have implications for the organization of space in the facility. In low or middle income country hospitals, a lounge near the labour ward was deemed necessary for companions to take short breaks [25]; in others providing a private space or a cubicle for the dyad of the labouring women and her companion was considered to be important for the success of the intervention [27, 39, 43, 44]. Crowding of shared labour rooms was a major concern in resource-constrained facilities [16, 43].

In facilities with shortage of nurses or midwives, the possibility of hiring and training unemployed or retired nurses and midwives was suggested [24, 26, 45] as well as training of lay companions [22, 43, 45] or doulas [23]. Clear communication with the companion about his/her duties was deemed necessary to reduce the negative providers’ perceptions about the role of the companion [43, 46].

Although none of the reviewed studies reported cost analysis, there were some discussion on expected cost implications in few of these studies. In high-income countries and among high-income populations, it is expected that the cost of hiring a doula will be covered by women themselves as very few hospital-based programmes offer that option [22]. In low-income settings, the involvement of family members as companions during childbirth is an inexpensive practice [16] however, the cost of transportation for volunteer companions need to be considered to sustain the programme at the facility [27]. In general, it is expected that the beneficial effect of a companion of choice at birth would be cost-effective for healthcare insurers and for hospitals as it reduces the financial costs of obstetric interventions such as epidural use and cesarean sections and the time spent on complications [16, 32].

## Discussion

Women reported positive experiences with having a companion of choice at birth across the different facility settings and country-income levels in the reviewed studies, regardless of the person providing the support. In facilities where male partners’ presence was accepted in addition to the female companion, an added value of the female companion was reported. Whereas the evidence indicates that companion of choice at birth is mostly an effective intervention when provided by persons who are not employed by the hospital [2], considerations should be given to the acceptance of doulas or family members in each cultural context as well as to the entailed costs for training or transportation.

We note the importance of the person providing the support in terms of increased women’s satisfaction with the birth experience. Only four studies reported on this outcome [7, 21, 23, 24]. Two studies that included lay female relatives of labouring women as companions reported an increased satisfaction of women during childbirth [21, 23]. The effectiveness of the intervention was attributed by the authors to the fact that the companions were related to women therefore more in tune with women’s needs [23] and to the positive influence of their presence on health care providers’ behaviours in terms of sharing of information related to the care given to the labouring woman [21]. Another study showing an increase in women’s satisfaction also reported the provision of information as a positive factor influencing women’s satisfaction, however in this case the support during childbirth was provided by midwives [7]. One trial in Mexico that used retired nurses as companions during childbirth reported no effect on women’s satisfaction [24]. Retired nurses were considered to be “de-sensitized” to women’s needs and less empathetic than lay companions [24].

Providers’ perceived the usefulness of the companions in facilities suffering from nurse and midwife shortage. In all of studies conducted in high or low income countries, providers carried negative attitudes towards the implementation of companion of choice at birth. They reported concerns about cross infection and crowding in labour wards as well as about the expected collaboration of women and of companions with the healthcare team and feared interferences with clinical decisions.

Other implementation barriers existed such as the allocation of resources within the facility and the organization of care to facilitate nurses or midwives continuous presence with the woman during labour and birth. Barriers in resource constrained environments related mainly to crowding and availability of space and privacy for women and their companions in the labour ward as well as to cultural preferences of the companion.

The implementation of this practice requires the commitment of the management of health care facilities to change institutional policies and to provide the appropriate physical space that respects women’s and their companion’s privacy. This could be informed by recent literature on the importance of supporting the supporters during childbirth through observing their interaction with the space [48]. While the evidence doesn’t indicate the necessity of providing training to the lay companion [2], there were different approaches used in the reviewed interventions and considerations need to be given to various modes of orienting the companion in order to improve the acceptance of the intervention mainly by the health care providers.

Influencing the attitudes of health care providers is necessary for the successful implementation of the intervention. This could be achieved through sensitization activities including the provision of evidence-based information, through minimizing system barriers such as to avoid overloading the staff and resolving issues of space and privacy, through affecting providers’ behaviours and through sharing of women’s positive experiences with this practice to motivate their participation. The latter can be achieved through the use of effective communication to clarify the role of the companion in the labour and delivery wards.

Given the improved health outcomes reported as well as the positive experiences of women with companion of choice at birth reported regardless of the person providing support across all studies, we had expected that the use of this practice in hospitals would increase facility births. However, no trials reported on the effect of this practice on this outcome. Two studies reported on women’s views reported that being denied of having a companion during childbirth can act as a reason for women opting to give birth at home [38, 39]. The need for such specific studies in those contexts where facility births are low would confirm if increased quality and increased satisfaction with services increases use of the services.

Implementation factors were mostly described in the qualitative studies identified for this review. These studies were descriptive in nature and focused on women’s experiences and views [37, 38, 41, 45], few also included the perspectives of health care providers and discussed system related factors important for implementation [39, 43, 46].

## Conclusion

More effort should be put in documenting and reporting implementation factors associated with interventions, by describing the context in terms of facility practices, providers’ attitudes, community culture and challenges, either within the reporting of the intervention outcomes or in separate publications. More importantly, future studies should use implementation research methods to develop and test companionship models in different facility settings and cultures. This will inform initiatives that aim at for scaling-up these models of care to a larger number of health care settings in their region or country.

Future research should also focus on measuring the costs pertaining to the training of personnel or the lay companion and to the changes in the physical space, as well as on the impact of companion of choice at birth on care seeking behavior for subsequent births.
